# Mindfulness for Motor and Nonmotor Dysfunctions in Parkinson's Disease

**DOI:** 10.1155/2016/7109052

**Published:** 2016-04-10

**Authors:** Nadeeka N. W. Dissanayaka, Farah Idu Jion, Nancy A. Pachana, John D. O'Sullivan, Rodney Marsh, Gerard J. Byrne, Paul Harnett

**Affiliations:** ^1^UQ Centre for Clinical Research, The University of Queensland, Brisbane, QLD 4029, Australia; ^2^School of Psychology, The University of Queensland, Brisbane, QLD 4067, Australia; ^3^Neurology Research Centre, Royal Brisbane & Women's Hospital, Brisbane, QLD 4029, Australia; ^4^School of Medicine, The University of Queensland, Royal Brisbane & Women's Hospital, Brisbane, QLD 4029, Australia; ^5^Mental Health Service, Royal Brisbane & Women's Hospital, Brisbane, QLD 4029, Australia

## Abstract

*Background*. Motor and nonmotor symptoms negatively influence Parkinson's disease (PD) patients' quality of life. Mindfulness interventions have been a recent focus in PD. The present study explores effectiveness of a manualized group mindfulness intervention tailored for PD in improving both motor and neuropsychiatric deficits in PD.* Methods*. Fourteen PD patients completed an 8-week mindfulness intervention that included 6 sessions. The Five Facet Mindfulness Questionnaire (FFMQ), Geriatric Anxiety Inventory, Hamilton Depression Rating Scale, PD Cognitive Rating Scale, Unified PD Rating Scale, PD Quality of Life Questionnaire, and Outcome Questionnaire (OQ-45) were administered before and after the intervention. Participants also completed the FFMQ-15 at each session. Gains at postassessment and at 6-month follow-up were compared to baseline using paired *t*-tests and Wilcoxon nonparametric tests.* Results*. A significant increase in FFMQ-Observe subscale, a reduction in anxiety, depression, and OQ-45 symptom distress, an increase in PDCRS-Subcortical scores, and an improvement in postural instability, gait, and rigidity motor symptoms were observed at postassessment. Gains for the PDCRS were sustained at follow-up.* Conclusion*. The mindfulness intervention tailored for PD is associated with reduced anxiety and depression and improved cognitive and motor functioning. A randomised controlled trial using a large sample of PD patients is warranted.

## 1. Introduction

Parkinson's disease (PD) is a chronic, progressive, incurable, complex, and disabling age-related disease. Classically, PD is characterised by abnormalities in movement; however, nonmotor symptoms including depression, anxiety, and cognitive decline are frequently experienced in PD [1–3]. Both motor and nonmotor symptoms negatively impact PD patients' quality of life. Over 50% of PD patients experience anxiety and depressive disorders [4]. The majority of PD patients develop mild cognitive impairment that may progress to dementia at advanced PD. The prevalence of dementia in advanced stage PD exceeds 80% [5]. At present, there are no effective therapies to treat anxiety or cognitive deficits in PD; very few randomised controlled psychotherapy trials for depression in PD have been attempted [6–8]. While Cognitive Behaviour Therapy (CBT) has been a popular psychotherapy method trialled in PD [6], there has been relatively little attention directed to mindfulness interventions in PD [9]. The present study explores the benefits of a manualized and tailored mindfulness group intervention to reduce anxiety and depressive symptoms in PD. The study also investigates the impact of the intervention on cognitive and motor symptoms in PD.

“Mindfulness” refers to the process of bringing awareness to moment-by-moment experience in a compassionate and nonjudgmental manner [10]. It can be further defined as a process of self-regulation of attention through increased awareness and recognition of mental events accompanied by a sense of curiosity, openness, and acceptance of one's experiences as they arise in the present moment [11]. The most frequently cited method of mindfulness training is the Mindfulness-Based Stress Reduction (MBSR) [12]. MBSR was originally designed to facilitate adaptation to stress of medical illness and to assist people in managing stress and pain [13]. Additionally, there are other mindfulness-based therapies such as the Mindfulness-Based Cognitive Therapy (MBCT), Dialectical Behaviour Therapy (DBT), and Acceptance and Commitment Therapy (ACT) [14]. Mindfulness has been adopted as an approach to increase awareness and respond skilfully to the mental processes that contribute to emotional distress and maladaptive behaviour [11]. Mindfulness allows individuals to expose themselves to all internal and external stimuli in an accepting rather than avoidant manner, provides the self with the ability to relook and respond to mental events for what they are without ascribing emotional valence to them to warrant reaction, and broadens the individual's repertoire of coping skills in difficult situations through increased self-awareness [15]. Individuals that undergo mindfulness training therefore increase their ability to observe, describe, act with awareness, be nonjudgmental of their inner experiences, and be nonreactive to their inner experience. These are the five facets of trait mindfulness [16].

A growing body of research has employed mindfulness for the enhancement of cognition and for the treatment of affective and anxiety disturbances [14, 17–19]. A recent review of mindfulness studies suggested that mindfulness-based therapy significantly improves selective attention, sustained attention, working memory capacity, and executive functioning [14]. Therefore, the technique of mindfulness addresses cognitive, affective, and anxiety disturbances that are common, progressive, and debilitative in persons with PD. Mindfulness interventions have been trialled with positive gains in several chronic diseases [20–22]. In PD, a qualitative study focused on MBCT has shown a number of benefits for patients including changing patterns of coping by reducing avoidance, changing stress and depression, and changing thoughts about thoughts, consolidating existing coping strategies in the context of loss, group support to socialise, share a common experience, learn, build confidence, and achieve a sense of social coherence, and identifying the duality between the psychological and physical experience of PD [23]. Subsequently, a quantitative controlled study by Pickut et al. (2015) [9] suggested an improvement in the Observe subscale of the Five Facet Mindfulness Questionnaire (FFMQ) and a reduction in motor disability, but no change in depression measured using the Beck Depression Inventory (BDI). Although their mindfulness protocol was based on MBSR, the study did not assess outcomes on anxiety. Another recent uncontrolled pilot study by Cash et al. (2016) [24] used a similar MBSR protocol and suggested improvements in FFMQ-Observe, FFMQ-Nonjudgment, and FFMQ-Nonreactivity subscales, depression, and cognitive functions including mental flexibility and complex attention. The intervention did not show significant improvement in anxiety measured by self-report screening Generalised Anxiety Disorder-9. This anxiety screen is not validated in PD and warrants further investigation.

Overall, previous mindfulness interventions trialled in PD patients show promise for further investigation of the benefits of mindfulness for both motor and nonmotor dysfunction in PD. The present study is an exploratory pilot study to examine the outcomes of a new manualized mindfulness intervention specifically tailored for persons with PD. The study uses a validated and recommended battery of assessments to investigate the effects of the new tailored mindfulness intervention on anxiety, depression, cognitive impairment, motor disability, and quality of life in PD immediately following the intervention and at 6-month follow-up. Qualitative assessments were also performed to further examine patients' views on their expectations and gains from the intervention.

## 2. Methods

### 2.1. Participants and Ethics

A convenience sample of PD patients was recruited from neurology outpatient clinics and Queensland Parkinson's disease database. Queensland Parkinson's project is a collaborative study of over 4000 PD patients and controls who have expressed interest in participating in PD research [2]. All PD patients had a diagnosis of idiopathic PD made by Movement Disorders Neurologists following the UK brain bank criteria [25]. Patients with dementia identified by the neurologist or scoring <24 in the Standardised Mini-Mental State Examination (SMMSE) were excluded [26]. The study excluded PD patients who have had functional neurosurgery such as deep brain stimulation. Written informed consent was obtained from all participants prior to commencing the study. The project was approved by the ethics committees of the University of Queensland and the Royal Brisbane & Women's Hospital.

### 2.2. Data Collection

Participants completed self-report questionnaires and were interviewed by an advanced postgraduate trainee who is a provisionally registered psychologist (2nd author) who completed clinician-rated measures. This data collection occurred a week prior to the mindfulness intervention. The intervention included 6 sessions, was conducted over 8 weeks, and was followed by postassessments conducted a week after the intervention. The postassessment included the same data collection as the baseline preassessment. Participants were followed up at six months from the postassessment to ascertain whether any gains they had received from the program were sustained. This follow-up assessment repeated the baseline measures and subsequently a telephone Mindfulness Booster session was delivered. This booster session was a refresher to the knowledge and skills covered in the intervention and encouraged continued mindfulness practice.

#### 2.2.1. Baseline, Postassessment, and 6-Month Follow-Up Measures

*Mindfulness*. The brief 15-item self-report Five Facet Mindfulness Questionnaire (FFMQ-15) was used to measure mindfulness. This is a short form of the 39-item FFMQ [16]. The 15-item version includes three items from each of the five subscales that had the highest factor loadings reported by Baer et al. [27]. Baer et al. [28] found that the alphas for the total mindfulness score of the abbreviated version (the sum of the 15 items) ranged from 0.80 to 0.85 when administered weekly over seven weeks. 

*Anxiety*. The self-report Geriatric Anxiety Inventory (GAI) was used to measure anxiety symptomology [29]. This is a validated scale in PD [30] and is recommended for use in PD [31]. 

*Depression*. The clinician-rated 17-item Hamilton Depression Rating Scale (HAM-D) [32] was used to measure depression. This is a validated and recommended scale to assess depression in PD [33]. We used a recommended “inclusive” approach when assessing depression where all symptoms were assessed individually regardless of the causal attribution [34]. 

*Cognitive Impairment*. Parkinson's Disease Cognitive Rating Scale (PDCRS) [35] was used to assess cognitive impairment. PDCRS is a recommended scale by the Movement Disorders Society. This scale was administered at the interview. 

*Motor Disability*. The interview also included the Movement Disorders Society Unified Parkinson's Disease Rating Scale (MDS-UPDRS) [36] for the assessment of the severity of PD and motor disability. Using the MDS-UPDRS, subscales of tremor and postural instability gait dysfunction (PIGD) were calculated following the previously published criteria [37]. 

*Quality of Life*. The self-report Parkinson's Disease Quality of Life Questionnaire (PDQ-39) [38] was used to measure quality of life. 

*Psychological Distress*. The Outcome Questionnaire (OQ-45) [39] was used to measure psychological distress. 

*Medication*. PD medication, antidepressants, and anxiolytics were recorded. A levodopa equivalent daily dose for PD medication was calculated following published criteria [40].

#### 2.2.2. Assessment Completed during Intervention Sessions

At each of the 6 intervention sessions, PD patients completed the brief 15-item FFMQ [28].

#### 2.2.3. Qualitative Assessment

At the end of each mindfulness session, participants were required to rate how useful they felt each session was to learning and applying mindfulness in their daily life. Participants were also required to list one key takeaway from each session, with the view of examining whether session objectives were successfully imparted. The overall feedback and suggestions were obtained at the postassessment. Participants were asked to provide comments regarding their experience of the program and their suggestions for future mindfulness research and intervention for individuals with Parkinson's disease.

### 2.3. Intervention

**Figure 1:**
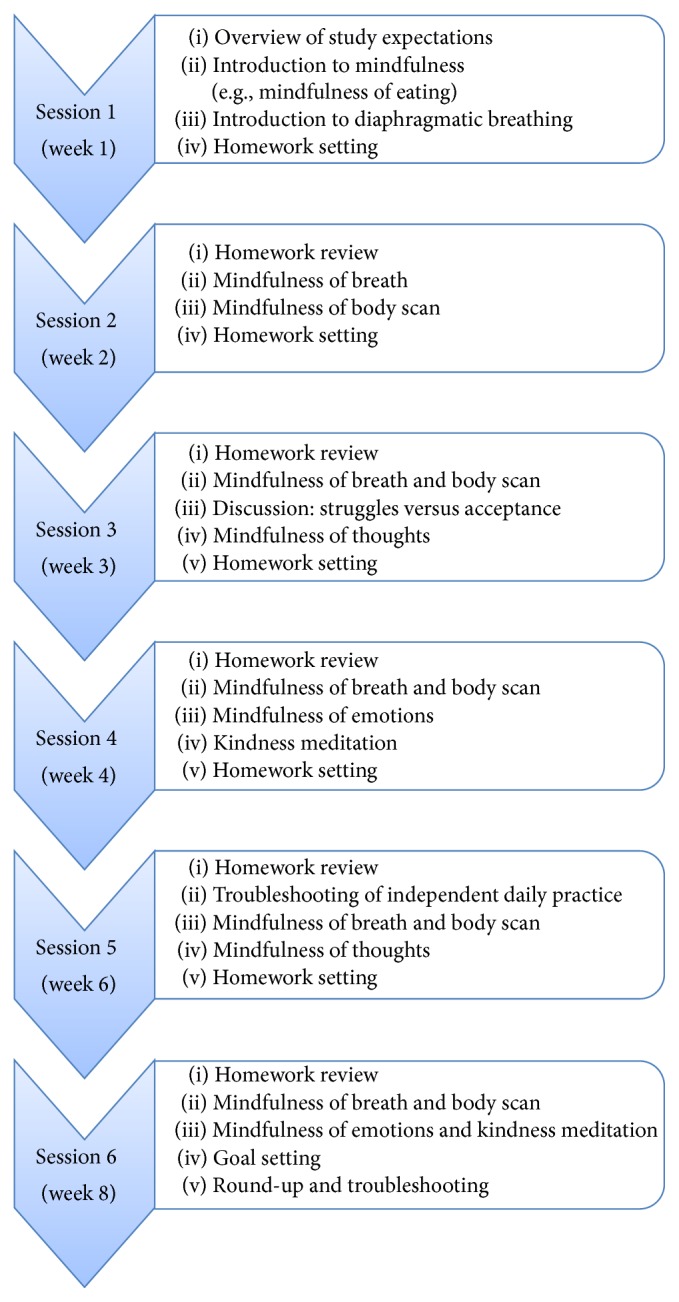
A summary of the mindfulness intervention.

The intervention was conducted in groups including 4-5 participants each. A manualized intervention protocol was developed for this study with content and exercises adapted from an existing MBSR resource for the general community [41]. The intervention introduced and developed mindfulness knowledge and skills in participants with a view to empowering participants to practice independently in their day-to-day lives. The content of sessions 1 to 4 addressed the five key concepts of mindfulness, observing, describing, acting with awareness, nonjudging of inner experience, and nonreactivity to inner experience [27], through the following formal sitting meditation exercises: mindfulness of eating, mindfulness of the breath, mindfulness of breath and body, body scan, mindfulness of thoughts, mindfulness of emotions, and kindness meditation. Group discussions in each session also covered strategies to incorporate mindfulness into daily activities (informal mindfulness practice) (Figure 1).

#### 2.3.1. Modifications to the Protocol Tailored for PD

The following modifications to the protocol were tailored for PD patients. Walking meditation was omitted for safety purposes given postural and gait instability issues in PD. The language and pace of instructions were also modified to make it easier for participants to grasp concepts, exercises, and discussions. Given the possibility of sensory deficits such as loss of smell or taste, facilitators exercised discretion and sensitivity when explaining tasks such as mindfulness of eating, emphasising that the key purpose was to notice, with whatever senses possible. Formal mindfulness exercises in each session were followed by debriefing and discussion of participant's reactions and experiences, including the impact of these exercises on their mental state and PD symptoms such as tremors, and suggestions to improve their practice. Metaphors and anecdotes were employed in these discussions to aid understanding of mindfulness concepts.

#### 2.3.2. Six Sessions of Mindfulness

The first session included an overview of the intervention, psychoeducation, introduction to mindfulness, introduction to mindfulness of diaphragmatic breathing, and setting homework. From the second session onward, participants were encouraged to share reflections of their independent home practice and to offer support and suggestions to the rest of the group and enhance their individual mindfulness practice under homework review. Any issues and barriers to practice were identified and addressed. Upon completion of the planned formal mindfulness meditation exercises in session and debriefing segments, time was set aside for participants to jot their homework goals for the week into their workbooks. These goals could include how often they would like to practice mindfulness or for how long. Alongside formal guided meditations, participants were encouraged to practice informal mindfulness, as a means to generalise and apply mindfulness to a broad range of daily activities including eating, walking, and showering. While the first four sessions of the intervention were conducted weekly, the final two sessions were conducted fortnightly to allow participants to gain confidence to practice their mindfulness skills independently after the end of the study. These two sessions were developed as “check points” and included an expanded homework review and also revisit of the mindfulness exercises. The final session comprised a final summary of content and skills learnt, as well as overall debriefing before closing with a mindfulness goal setting segment where participants noted their aims to live life mindfully. The weekly session schedule is summarised in Figure 1.

#### 2.3.3. Homework

All participants were encouraged to practice mindfulness for a total of 45 minutes a day (or 315 minutes a week), comprising both formal and informal practice. While participants were urged to practice the formal meditations covered in their most recent sessions each week, they were allowed to select exercises that resonated well with them. Participants had to log their practice in their workbooks, rating the frequency, duration, and quality of practice for each instance.

### 2.4. Facilitators and Training

The workshops were run by six provisionally registered clinical psychologists undergoing advanced postgraduate training at the University of Queensland. Two facilitators (group leaders) were assigned to each group. This was to ensure that as one facilitator explained and read through the script for the mindfulness exercises, the other facilitator could take notes of participants' physical reactions and be available to respond to any difficulties that may arise. The presence of a cofacilitator also ensured that “blind spots” in facilitation were addressed. All facilitators followed the manualized intervention protocol, had previously been trained using a similar mindfulness intervention protocol, and had facilitated groups for the general population [41]. To ensure fidelity and that the protocol was adhered to, all facilitators were provided with a checklist of content and skills to be covered in meeting the session's goals and were required to complete a facilitator feedback sheet outlining whether they were able to meet the session's goals. Facilitators were also required to note their overall rating of the session and to provide their clinical impressions of how the group had responded to each session. The main researcher examined these logs weekly to ensure that all goals for each session were met. Regular supervision by registered clinical psychologists at the start, middle, and end of the study period was provided and specialised training in the area of PD was provided. This included understanding the physical symptomology of PD which may impact the facilitators' delivery of mindfulness exercises. During these group supervision meetings, facilitators were also given the opportunity to highlight any observations or difficulties that they encountered while working with their group and debrief their experiences.

### 2.5. Data Analysis

To determine changes in outcome variables between preintervention, postintervention, and 6-month follow-up, paired-samples *t*-tests were conducted. A test of normality was conducted on all outcome measures using the Shapiro-Wilk test. The data distributions that were identified as nonnormal were transformed by square root and reflected accordingly if negatively skewed. Data distributions that were unable to be corrected for normality even after the transformation and the removal of outliers were treated as nonnormal distributions and analysed using nonparametric statistics (Wilcoxon signed-ranks test). Repeated measures Analysis of Variance (ANOVA) within-subjects statistic was also employed when assessing the change for more than 2 time points of assessment such as assessing the change in trait mindfulness. For all procedures, alpha was set at 0.05 (two-tailed). Bonferroni corrections for multiple comparisons were not performed due to the small sample size and the exploratory nature of this pilot study. Statistical Package for the Social Sciences (SPSS Version 21) was used to perform all statistical analyses.

## 3. Results

### 3.1. Study Sample Characteristics and Attrition

Seventeen PD patients who met the study criteria completed preintervention assessments. Of these, 3 male participants withdrew, and therefore 14 individuals completed postintervention assessments (an attrition rate of 18%). Two of the participants withdrew before the commencement of the intervention, one due to a back injury and the other because of the inconvenience and cost of travel to the study location. The third withdrew after attending the first mindfulness session as he could not manage the anxiety he experienced being with a group of people.

Of the 14 participants who completed postintervention assessments, 9 were male (64.3%) and 5 were female (35.7%). Their age ranged between 52 and 78 years (mean: 66 years, SD = 7.43). The majority of the participants reported having a post-high school qualification (*n* = 10) that included postgraduate studies or a professional diploma, while two participants completed high school and two participants had less than high school qualification. The majority of the participants (*n* = 11) were married while the remaining three participants were widowed or divorced. The mean number of years since PD diagnosis was 4.50 (SD = 3.01). All participants reported taking their neurologist-prescribed PD-specific levodopa medication daily (mean L-dopa equivalent dosage in milligrams = 468, SD = 293). Six of the 14 participants (42.9%) had prior exposure to activities similar to mindfulness in the form of either meditation, yoga, or pilates. At baseline, two participants were actively practicing informal mindfulness (deliberate attention to their environment) while another attended pilates once a week. Participants expectations from the intervention included (a) better management of stress, anxiety, and mood (57.1%), (b) better management of PD-specific physical symptoms such as tremors (28.6%), (c) greater self-acceptance, self-awareness, and personal growth (42.3%), and (d) improving concentration and focus (21.4%).

At 6 months, 6 participants were lost to follow-up (final attrition rate is 53%). Therefore, follow-up assessments and booster sessions were conducted in 8 participants out of the 14 participants who completed postassessments.

### 3.2. Mindfulness Subscales

Table 1 presents the means and standard deviation and results of the *t*-tests carried out to assess change between pre- and postintervention. Total mindfulness scores on the FFMQ-15 at postintervention (M = 54.1, SD = 6.03, *p* = 0.05) just failed to reach a clinically significant improvement over the preintervention scores (M = 50.1, SD = 4.67). At follow-up, the total mindfulness score (M = 54.9, SD = 8.61) was not significantly higher than preintervention score. Looking at the subscale scores, a statistically significant increase between pre- and postintervention was found only for the FFMQ-15-Observing subscale (*t* = 2.44, *p* = 0.03), although the follow-up score was not significantly higher than the preintervention score.

Table 2 presents the weekly scores of the FFMQ-15. There was a reduction in the total mindfulness score at week 1 compared to the preintervention score. However, after the program started, the total FFMQ-15 score steadily increased. The scores at sessions 5 and 6 and postintervention were all significantly higher than session 1 score.

### 3.3. Anxiety and Depression

At postintervention, the GAI (*Z* = −2.20, *p* = 0.03) and HAM-D (*t* = 2.20, *p* = 0.04) scores were significantly reduced compared to baseline (Table 1). The number of participants who met clinical criteria for anxiety disorder using a validated optimal cut-off of 6/7 in the GAI [30] decreased from six individuals at preintervention to three at postintervention. The frequency of participants who met clinical criteria for depression using a validated optimal cut-off of 12/13 in the HAM-D [42] decreased from three individuals at preintervention to two individuals at postintervention.

Reliability change index (RCI) statistics were used to identify the number of individuals who demonstrated clinically significant change in the GAI and HAM-D [43]. The RCI is a statistical measure that can determine whether the change observed at postintervention compared to the preintervention for an individual is greater than a level that is unlikely to occur due to chance. Individuals exceeding a threshold RCI of 1.96 will show clinically significant change, and this is another method of determining the outcomes of an intervention with small sample sizes. Three (21%) PD patents showed a clinically significant reduction in anxiety as measured by the GAI and four (29%) showed a clinically significant reduction in depression as measured by the HAM-D.

There were no significant differences in the GAI scores at follow-up (M = 4.75, SD = 6.14, Mdn = 1.00) from postintervention (M = 3.25, SD = 3.96, Mdn = 2.00, *Z* = −0.18, *p* = 0.85) or from baseline (M = 3.88, SD = 4.94, Mdn = 1.00, *Z* = −1.29, *p* = 0.20). Based on the GAI cut-off score, out of 8 participants who completed the follow-up, at baseline, there were 3 individuals meeting the clinical criteria for anxiety, and this was reduced to 2 at postintervention but increased back to 3 at follow-up assessment. RCI analysis suggested clinically significant improvement in anxiety in 2 individuals at postassessment and 1 individual at follow-up compared to baseline.

There was a significant increase in the HAM-D at follow-up (M = 6.25, SD = 4.71) from postintervention (M = 3.38, SD = 3.20, *t*(7) = −0.285, *p* = 0.025, *d* = −0.11). There was no significant difference between follow-up and baseline (M = 6.63, SD = 4.81). Based on the HAM-D cut-off score, out of 8 participants, 1 met clinical criteria for depression at baseline and none did at postassessment, but this increased back to 1 participant at the follow-up. RCI analysis suggested clinically significant improvement in depression in 2 individuals at postassessment and 1 individual at follow-up compared to baseline.

### 3.4. Cognitive Functioning

The total PDCRS score was significantly higher at postintervention compared to the preintervention (*t* = −3.59, *p* < 0.01). There was no significant change in the PDCRS-Cortical score although the PDCRS-Subcortical score demonstrated a significant improvement (*t* = −3.63, *p* < 0.01). Within the subcortical subscale, items of working memory, alternating verbal fluency, and action verbal fluency showed significant improvement following the intervention compared to the baseline (Table 1).

The total PDCRS score did not differ between follow-up (M = 104, SD = 18.5) and postintervention (M = 102, SD = 18.9). However, a significant increase in the PDCRS total scores was observed at follow-up compared to baseline (M = 93.6, SD = 22.6, *t*(6) = −4.78, *p* = 0.0013).

There was also a significant increase in the subcortical subscale of the PD-CRS at follow-up (M = 74.8, SD = 18.0) compared to baseline (M = 62.3, SD = 20.0, *t*(6) = −5.84, *p* = 0.001, *d* = −2.38). There was no significant difference between follow-up and postintervention PDCRS-Subcortical scores (M = 73.0, SD = 17.4).

### 3.5. Motor Disability

The MDS-UPDRS total score or the MDS-UPDRS-III-Motor disability score did not show significant differences between pre- and postintervention; however, the PIGD subscale demonstrated a significant improvement at postintervention (*t* = 3.03, *p* = 0.01) compared to the baseline.

There was no significant difference in scores on all MDS-UPDRS subscales at follow-up from postassessment or from baseline. However, there was a significantly higher score in the rigidity item at follow-up (Mdn = 2.00) from postassessment (Mdn = 0.50, *Z* = −2.04, *p* = 0.041, *d* = −0.77), with rigidity scores deteriorating.

### 3.6. Quality of Life

No significant improvement in the PDQ-39 was observed in scores between preintervention (M = 22.8, SD = 14.8) and postintervention (M = 17.8, SD = 13.3) or between preintervention and follow-up (M = 17.8, SD = 12.6). There were also no significant differences in any of the eight PDQ-39 subscales between preintervention and postintervention (Table 1).

### 3.7. Psychological Distress

The OQ-45-Symptom Distress subscale score was significantly lower at postintervention compared to the baseline (*t* = 2.97, *p* = 0.01). However, at follow-up, a statistically significant increase in this distress score compared to baseline was observed (M = 7.88, SD = 4.19, *t*(7) = −6.04, *p* = 0.001).

### 3.8. Homework Compliance, Quality of Independent Practice, and Barriers to Independent Practice

Out of 14 participants, 8 individuals completed at least 90% of their logbook requirement with only 6 of these participants consistently documenting their practice weekly over the course of the program. Two participants did not complete their logbooks at all. The remaining participants documented their practice sporadically. Participants cited their reasons for not recording their practice consistently as follows: (i) it was too cumbersome to do so and (ii) their practice was incidental and they did not have their workbooks when practicing. Other reasons included that one participant had difficulty writing due to PD while another participant had injured his back. Notwithstanding, according to facilitators' records, participants had verbally reported practicing mindfulness outside of sessions and that this was evident based on the content of discussions during the homework review segment at the beginning of each group meeting. Therefore, the information obtained from the log was not completely representative of homework compliance. There was also great variability in the frequency and duration of practice per week by participants.

Based on the completed homework logs, a repeated measures ANOVA was conducted to assess any significant change in number of hours of home practice over the course of the intervention. There was a significant increase in the number of hours participants reported practicing mindfulness since their first session (*F*(4,20) = 3.07, *p* = 0.04, *ηp* ^2^ = 0.380), with pairwise comparisons revealing that the mean number of hours practiced per week significantly increased from 176 hours (SD = 31.0) at week 1 to 327 hours by week 5 (SD = 63.3, *p* = 0.027). There was also a significant increase in the quality of their practice compared to week 1, with pairwise comparisons revealing that quality ratings increased from an average of 6 (SD = 1.05) to an average of 7.33 (SD = 0.61, *F*(4,20) = 4.69, *p* = 0.008, *ηp* ^2^ = 0.484). There was no significant difference in the frequency of practice over the weeks, with participants practicing an average of between 8 and 11 times per week of both formal and informal mindfulness.

There was no significant correlation between homework compliance in terms of the number of minutes spent on mindfulness each week, quality of practice, and frequency of practice on scores on the FFMQ-15. Linear regression analyses were also conducted to explore the predictive ability of homework compliance and FFMQ-15 scores, but results yielded no significant effects.

By the postintervention interview, participants reported that they favoured informal mindfulness practice. All participants noted that they practiced informal mindfulness almost daily, while about 57% noted practicing at least twice a week of formal practice. However, there was a marked decline in the practice of mindfulness by the 6-month follow-up interview. Only one of the eight participants who attended the follow-up sessions reported that they practiced formal mindfulness almost on a daily basis, while three participants reported practicing mindfulness once a fortnight or when they feel stressed. Participants continued to favour informal mindfulness practice at follow-up, with 75% of the participants at follow-up reporting practicing informal mindfulness at least five times a week.

### 3.9. Participant Overall Feedback and Suggestions

All 14 (100%) participants reported that they enjoyed the mindfulness program, while 78.6% fully agreed that their expectations were completely met. All participants agreed that they would recommend the program to others. Five main themes emerged when participants were asked how they felt they had benefitted from the program. They were (i) increased awareness and observational skills, (ii) better stress and anxiety management, (iii) improved psychological and emotional wellbeing, (iv) physical improvement, and (v) psychological improvement. 

*(i) Increased Awareness and Observational Skills*. The majority of participants noted that they were more observant of their inner and outer environments. Participants had noted being more aware of their day-to-day actions while eating, driving, showering, or playing with grandchildren. They also reported an increase in their observational skills, such as while going for walks, as they attend to their surroundings better after the program (such as listening to birds, noticing the weather). Two participants noted how conversations with others had improved due to their ability to stay in the moment and listen to the other party. Participants also reported having greater awareness of their mental processes and emotional states. Two participants described how they were able to recognise their anxiety better following the program and therefore manage accordingly using the breathing techniques learnt in the program. 

*(ii) Better Stress and Anxiety Management*. Participants cited that the program had empowered them with a new tool to better manage and cope with day-to-day stressors. Participants had also reported that the breathing exercises learnt had enabled them to relax better. One participant noted that their spouse had noticed that they managed their life better. One participant described this as follows:

*It's (the Mindfulness skills) given me an out. I can do it anytime, anywhere and anyplace. I used to give up whenever I get stressed but now I just close my eyes and take it easy.*

Several participants also noted an increased ability to manage distressing thoughts and being better able to let go of emotionally charged thoughts. One participant described their experience as follows:

*I've learnt skills to relax and clear my mind. Focusing on breathing anchored me to the present instead of all the muddling up in my head. *

Another participant noted the following after the program:

*I'm not affected by thoughts about the future too much. I find myself less worried. I am less future-focused.*

Another participant described the following:

*I've learnt that I have permission to let my thoughts wonder. I accept it. Focus on present rather than be pre-occupied.*

*(iii) Psychological and Emotional Wellbeing*. Participants noted that they experienced an improvement in their mood following the end of the program. Two participants noted that they felt less irritable and were more tolerant of people. Another participant described how they were happier and that they developed a closer relationship with their spouse, having developing a more positive outlook at home from the program. Another participant described the improvement in their happiness with mindfulness as follows:

*Whenever I meditate, it is one of the few times that I feel a sense of joy flooding through the system…which I hadn't felt in a long time. *

Participants also noted an improvement in their self-acceptance. One participant described having “*found peace within myself*” while another noted that their main takeaway from the program was to be nonjudgmental. Another participant described this as follows: “*The key takeaway (from this program) is that I can be nice to myself…not to be so hard on myself. If something (bad) happens and I can't change it, I have to deal with it. I became aware of how critical I was of myself. It made me aware that I need to take care of myself.”*

*(iv) Physical Improvement*. Eight participants noted an improvement in their physical wellbeing. Two participants reported that they felt physically more active following the program. Two participants noted that the breathing components of mindfulness exercises had helped them breathe better and also helped them with stiffness and physical discomfort by “*breathing into the pain.*” One participant mentioned that their blood pressure would lower by 10–15 points after each meditation.

Three participants described that their tremors would reduce after formal meditation. One participant reported that this improvement would remain even 10 minutes after the meditation:

*When I meditate (Mindfulness of Breath and Body), my tremors go down to a zero and stays like that for a while…emotions trigger my tremors so taking myself out through meditation helps. *

The meditation exercises cited by these three participants in helping with their tremors included mindfulness of the breath and body, the body scan, and mindfulness of thoughts. A participant described how they would also use breathing as a tool to manage their tremor:* “I noticed the shaking coming on and I did deep breathing and the shaking was gone.”*

*(v) Cognitive Improvement*. Five participants mentioned an improvement in their attention and concentration following the program. One participant also noted the immediate effects of practicing mindfulness as follows: “*After meditation, my vision is clearer and my overall senses and attention improves.*”

## 4. Discussion

This study was an exploratory evaluation of an 8-week, six-session group mindfulness-based training program for adults diagnosed with PD and was the first to explore outcomes at 6 month follow-up. The present study also included qualitative information about the mindfulness program.

The study showed a significant increase in the Five Facet Mindfulness Questionnaire-Observing subscale, a reduction in anxiety and depression scores, an improvement in cognition, a decrease in PIGD symptomatology, and a reduction in symptom distress observed from the OQ-45 immediately following the intervention. To date, there have been two other quantitative studies examining the effects of a similar mindfulness intervention in PD [9, 24]. In line with previous studies, the present study demonstrated an improvement in the trait mindfulness Observe subscale. However, unlike the study by Cash et al. (2016), we did not find a significant change in the other subscales measuring different facets of mindfulness or the overall trait mindfulness score between baseline and postintervention assessment. However, compared to session 1, the present study observed overall gains in the five-facet trait mindfulness at sessions 5 and 6 and postintervention. The mindfulness protocol used in this study is a modified MBSR protocol [44] previously trialled in the general population, which have also suggested positive overall gains in the trait mindfulness [41].

Importantly, the present study is the first to demonstrate that the new tailored mindfulness intervention is effective for the management of anxiety in PD. Anxiety is a common complication in PD without effective treatment [4, 8]. Over 50% of PD patients experience significant anxiety, and it negatively impacts patients' quality of life [1, 45]. While depression has been the focus of many psychotherapy studies, anxiety is always examined secondary to depression [46]. However, 75% of PD patients experience anxiety without concomitant depression [47]. Therefore, exploring treatment strategies for anxiety in PD is vital for the management of anxiety disorders in PD. The present study provides preliminary indications that a tailored MBSR intervention may reduce anxiety in PD. While general anxiety rating scales are criticised as inappropriate for use in PD, the present study carefully selected a validated and recommended anxiety rating scale, the GAI, to assess anxiety in this PD sample [30, 31]. Although at baseline the mean GAI score was lower than the optimal cut-off value of 6/7 for a clinically significant anxiety disorder, 43% of the sample (6 out of 14) scored >6 suggesting the presence of a clinically significant anxiety disorder at baseline. At postintervention, a 50% reduction in anxiety disorders was found; that is, at postassessment, 21% (3 out of 14) scored above threshold for an anxiety disorder. We confirmed these results further by performing RCI analysis and suggested that 3 individuals had a clinically significant change in anxiety due to the intervention.

At baseline, the frequency of patients who met clinical criteria for depressive disorder based on scoring higher than 12 in the HAM-D was lower than of those who met threshold for anxiety disorder. However, our results suggested that the mindfulness intervention can also be effective to reduce symptoms of depression. Our results are in line with the previous mindfulness study by Cash et al. (2016) [24], but contrary to the previous study by Pickut et al. (2015) [9], where no change in depression was observed using the BDI. While the self-report BDI is a validated instrument in PD [33], there has been some criticism for its use in PD [48]. The present study employed a well-validated clinician report and recommended HAM-D to assess depression in PD [33]. We showed 4 individuals with clinically significant change in depression due to the intervention. Our results are in line with the literature suggesting that mindfulness is an effective intervention to reduce both anxiety and depression in general [14, 17–19].

The present study also explored the benefits of a mindfulness intervention on cognitive functioning in PD. Cognitive decline is a common complication in PD. It has been suggested that cognitive deficits in PD can be identified as early as at the time of initial diagnosis [49]. In many cases, PD patients shift from mild cognitive impairment (MCI) to dementia at advanced PD, and it has been shown that dementia can occur at prevalence as high as 80% in PD [5, 50]. Despite the high prevalence of cognitive impairment in PD, there are no effective treatments, and developing safe and targeted nonpharmacological strategies is essential to combat cognitive deficits in PD. Our exploratory analysis suggested that the new tailored mindfulness protocol trialled in the present study may significantly improve cognitive functioning in PD. In particular, the performance on subcortical cognitive domains significantly improved following the intervention. Further examination of PDCRS items revealed that working memory and verbal and action word fluency tasks are significantly improved following the intervention. These findings are consistent with findings of the previous mindfulness for PD study [24] and existing research examining mindfulness meditation and cognition in the general population [14]. The findings are also similar to the improvement in cognition function found in cognitive rehabilitation program previously used in PD [51, 52], suggesting that the mindfulness intervention could itself be a form of cognitive training program. Therefore, mindfulness can be used in PD patients who poorly perform in cognitive tasks. Interestingly, recent studies have demonstrated that deficits in verbal fluency and executive functioning are early cognitive impairment in PD [53, 54], and the present study provides preliminary evidence suggesting that the mindfulness training may reduce such early cognitive deficits observed in PD. To reduce participant fatigue from completing lengthy assessments and the exploratory nature of the study, we limited our cognitive assessment to level 1 screen of cognitive decline using the PDCRS. The results can be further examined using extensive level 2 screen of cognitive functioning [55] in future study.

In comparison to the previous mindfulness study in PD [9], the present study did not show a change in total motor disability score assessed by the MDS-UPDRS III. However, further explorations suggested that the symptoms of PIGD, but not tremor, can be reduced following the intervention. Similarly, the present study demonstrated significant improvements in the MDS-UPDRS gait, posture, and rigidity items at postintervention. Similar to previous studies [9, 24], the present study did not show overall improvement in the PD health related quality of life measured by the PDQ-39 following the intervention. However, the results suggested a reduction in the reporting of symptom distress assessed using the OQ-45-Symptom Distress subscale.

Compared to previous mindfulness studies in PD [9, 24], inclusion of a 6-month follow-up assessment and a booster session was a novel aspect of the present study. Although the mindfulness program showed significant gains in anxiety and depression immediately after intervention, they were not sustained at follow-up in our small sample size of 8 patients who completed follow-up assessments. This may suggest that continuous and sustained active engagement received during the intervention is required for long-term benefits of the mindfulness for depression and anxiety. The progressive neurodegeneration in PD may increase vulnerability to depression and anxiety in PD [1, 2, 45], and therefore within a 6-month period patients may develop depression and anxiety. As expected, at 6 months, deterioration in motor function and increased reporting of symptom distress were observed. However, interestingly, results for subcortical cognitive gains remained at 6-month follow-up. Further examinations of these cognitive gains warrant future study.

Overall, improvement in both motor and neuropsychiatric deficits in PD immediately following the mindfulness intervention is in line with the recent neurobiological changes illustrated following a similar mindfulness training program in PD [56]. This previous study examining structural brain changes after intervention demonstrated an increase in grey matter density in brain areas responsible for emotion, anxiety, and cognitive and motor functioning relevant to PD. Investigating the impact of functional changes in the brain and associated functional brain networks corresponding to these structural changes and behavioural observations of the present study will be of interest for the future.

No change was observed on Parkinson's Disease Quality of Life Questionnaire. This is not surprising given that majority of questions on this scale relate to aspects of functioning that would not be expected to change in response to a short-term mindfulness program, such as mobility in the patients' home and in public. It might be that longer-term benefits of mindfulness may be observed if sufferers of PD are able to cultivate nonjudgmental acceptance of aspects of their condition. The benefits of mindfulness-based interventions for the management of chronic pain suggest that techniques such as mindfulness and yoga that encourage awareness and exploration of the body's range of movement may contribute to a reduction in the fear of movement and fear of pain/reinjury [57]. It is also possible that combining mindfulness-based interventions with other nonpharmaceutical interventions such as exercise and dance training might help to improve quality of life and address the issue of sustainability of change. The long-term benefits of mindfulness are likely to be greater in people who maintain a regular mindfulness practice. A recent systematic review of the impact of dance and exercise classes described studies that suggest that a proportion of participants who participated in dance classes enrolled in further dance classes on completion of the research trials [58]. This was not true for participants who had participated in exercise classes. Future research could usefully investigate whether combining mindfulness-based techniques with dance and other activities may motivate people to maintain a practice and whether this leads to an increase and sustained improvement in quality of life.

Despite positive gains on both motor and nonmotor impairment in PD observed following the mindfulness intervention, there are a number of limitations of this study worthy of note. The study was performed as an exploratory analysis testing a newly developed manualized mindfulness protocol that is tailored for PD. This pilot analysis did not include a controlled sample and also had a small sample size. The sample of 14 patients at postassessment and 8 at follow-up was not adequate to perform alpha reductions for multiple tests and was a limitation of this study. Although our attrition rate at postassessment (18%) was lower than the previous study (25%) [24], the attrition at follow-up was high (52%). The progressive nature of PD may increase disability over time and may result in a greater number of participants withdrawing from the study at follow-up time points. Moreover, without a controlled comparison, we cannot evaluate the placebo effect of the results. Exclusion of patients with dementia and those who have had functional neurosurgery such as deep brain stimulation is another limitation of the study. Future mindfulness studies can include persons who have had neurosurgery. Impact of the mindfulness intervention on PD persons with dementia can be evaluated in the future by modifying the protocol (e.g., individualised sessions) and involving caregivers in the mindfulness intervention.

## 5. Conclusion

In conclusion, the present exploratory study provides evidence that the tailored mindfulness intervention reduces anxiety and depression and enhances cognitive and motor functioning in PD. These positive gains of the intervention were revealed both qualitatively and quantitatively. These promising results warrant future investigations in a randomised controlled trial using a large sample of PD patients.

## Figures and Tables

**Table 1:** A comparison of outcome measures between baseline (pre-) and postassessment.

Measure	Pre	Post	Test		
	Mean (SD)	Mean (SD)	*t*	*Z*	*p*
*Mindfulness*					
FFMQ-15	50.30 (4.36)	53.8 (6.37)	−2.16		0.05
FFMQ-15-Observing	9.67 (2.93)	11.20 (2.26)	2.44		0.03^*∗*^
*Anxiety*					
GAI^a^	4.85 (5.14)	2.93 (3.67)		−2.20	0.03^*∗*^
*Depression*					
HAM-D^b^	2.49 (1.37)	1.66 (1.32)	2.20		0.04^*∗*^
*Cognition*					
PDCRS-Total	94.9 (21.1)	102 (17.2)	−3.59		<0.01^*∗*^
PDCRS-Cortical^a^	28.9 (1.51)	29.3 (1.55)		−1.00	0.32
PDCRS-Subcortical	66.2 (19.2)	73.0 (15.2)	−3.63		<0.01^*∗*^
PDCRS-Working Memory^b^	1.54 (0.62)	2.71 (0.24)	−0.40		<0.01^*∗*^
PDCRS-Alternating Verbal Fluency	11.4 (4.41)	12.9 (4.20)	−2.50		0.03^*∗*^
PDCRS-Action Verbal Fluency	14.7 (6.71)	17.5 (6.96)	−3.36		<0.01^*∗*^
*Motor disability*					
MDS-UPDRS-Total	38.9 (11.0)	35.2 (14.6)	1.22		0.25
MDS-UPDRS III-Motor score^a^	21.1 (6.28)	20.3 (8.26)		−0.69	0.49
MDS-UPDRS-Tremor	7.43 (5.17)	7.43 (4.50)	0.00		1.00
MDS-UPDRS-PIGD^b^	1.36 (0.62)	1.05 (0.65)	3.03		0.01^*∗*^
Gait (item 3.10)^a^	1.07 (0.62)	0.64 (0.50)		−2.12	0.03^*∗*^
Posture (item 3.12)^a^	0.50 (0.85)	0.29 (0.61)		−1.73	0.08
Bradykinesia (item 3.14)^a^	0.86 (0.86)	1.07 (0.72)		−1.00	0.32
Tremor (items 3.15 to 3.18)	6.14 (4.35)	6.50 (4.05)	−0.41		0.69
Rigidity (items 3.3a to 3.3e)^a^	2.71 (1.90)	1.71 (1.33)		−2.17	0.03^*∗*^
*Quality of life*					
PDQ-39	25.2 (15.5)	22.4 (15.5)	1.31		0.22
Mobility	12.5 (11.2)	10.8 (10.7)	0.81		0.43
Activities of Daily Living^a^	15.8 (13.8)	13.8 (12.7)		−0.95	0.34
Emotional wellbeing	18.3 (17.5)	16.0 (13.7)	1.05		0.32
Stigma^a^	15.2 (19.7)	12.8 (16.9)		−1.39	0.16
Social Support^a^	12.5 (15.9)	12.8 (16.9)		−0.28	0.80
Cognition^b^	3.83 (1.98)	3.33 (2.49)	1.01		0.33
Communication^a^	18.5 (12.7)	14.1 (19.1)		−1.38	0.17
Bodily Discomfort	23.1 (20.5)	19.9 (14.3)	0.73		0.48
*Psychological wellbeing*					
OQ-45-Total	43.2 (16.0)	36.3 (21.6)	1.83		0.09
OQ-45-Symptom Distress	26.2 (9.97)	20.3 (13.0)	2.97		0.01^*∗*^

^a^Nonparametric distribution Wilcoxon; ^b^transformed by square root. PIGD: postural instability gait dysfunction; ^*∗*^statistical significance *p* < 0.05. FFMQ: Five Facet Mindfulness Questionnaire; GAI: Geriatric Anxiety Inventory; HAM-D: Hamilton Depression Rating Scale; PDCRS = Parkinson's Disease Cognitive Rating Scale; MDS-UPDRS: Movement Disorders Society Unified Parkinson's Disease Rating Scale; PDQ: Parkinson's Disease Quality of Life Questionnaire; OQ: Outcome Questionnaire.

**Table 2:** Weekly change in overall mindfulness scores.

Session	FFMQ-15 total score
	Mean (SD)
Preintervention	50.0 (4.35)
1	46.1 (6.78)
2	47.6 (5.46)
3	47.2 (8.50)
4	49.7 (8.02)
5	51.4 (8.10)^*∗*^
6	52.4 (6.23)^*∗*^
Postintervention	53.3 (6.38)^*∗*^

^*∗*^Significantly different compared to week 1 at *p* < 0.05.
